# Steroid receptor coactivators in Treg and Th17 cell biology and function

**DOI:** 10.3389/fimmu.2024.1389041

**Published:** 2024-04-18

**Authors:** Yosi Gilad, Ortal Shimon, Sang Jun Han, David M. Lonard, Bert W. O’Malley

**Affiliations:** ^1^ Department of Molecular and Cellular Biology, Baylor College of Medicine, Houston, TX, United States; ^2^ CoRegen, Inc., Baylor College of Medicine, Houston, TX, United States; ^3^ Nuclear Receptor, Transcription and Chromatin Biology Program, Dan L. Duncan Cancer Center, Baylor College of Medicine, Houston, TX, United States

**Keywords:** steroid receptor coactivators (SRCs), nuclear coactivators (NCoAs), Th17 cells, Treg cells, cell therapy

## Abstract

Steroid receptor coactivators (SRCs) are master regulators of transcription that play key roles in human physiology and pathology. SRCs are particularly important for the regulation of the immune system with major roles in lymphocyte fate determination and function, macrophage activity, regulation of nuclear factor κB (NF-κB) transcriptional activity and other immune system biology. The three members of the p160 SRC family comprise a network of immune-regulatory proteins that can function independently or act in synergy with each other, and compensate for - or moderate - the activity of other SRCs. Recent evidence indicates that the SRCs are key participants in governing numerous aspects of CD4^+^ T cell biology. Here we review findings that establish the SRCs as essential regulators of regulatory T cells (Tregs) and T helper 17 (Th17) cells, with a focus on their crucial roles in Treg immunity in cancer and Treg-Th17 cell phenotypic plasticity.

## 1 Introduction

CD4^+^ T cells are a major subset of T lymphocytes that are further divided into distinct subpopulations which play various pivotal roles in immunity. CD4^+^ T cells coordinate both innate and adaptive immune responses through their interaction with and modulation of other immune cells or by their direct action via cytokine signaling (1). However, CD4^+^ T cells function not only as helpers for other immune cells and communicators of immune response; different subtypes of CD4^+^ T cells have direct cytotoxic activity while others act as suppressors of inflammation (1, 2). The wide range of functionality seen in the diverse subclassifications of CD4^+^ T cells is controlled by dynamic transcriptional regulation at different stages during their development, as well as through adaptation of specific transcriptional programs as these cells react to cues in their microenvironment (3).

Nuclear receptor coregulators (coactivators and corepressors) are a large family of proteins that regulate transcription not by a direct interaction with the DNA, but rather by binding to nuclear receptors (NRs) and other transcription factors to promote the assembly of chromatin remodeling factors and transcriptional machinery (4, 5). Since the discovery and identification of the first nuclear coregulator, SRC-1, in 1995 (6) ~ 300 coregulators have been identified and characterized as critically important regulators of gene expression (7). SRCs represent a group of three homologous nuclear coregulators that share a high degree of structural similarity, and all have a molecular weight of approximately 160 kDa. All three SRCs play important roles in major physiological and pathological processes such as fertility, metabolism, wound healing, development, immunology and oncogenesis (8–10). The importance of the SRCs in immunology is understudied but is evidenced by studies that show their participation in fate determination and function of lymphocytes and macrophages, regulation of lymphoproliferation and the ability to interact with and coordinate the transcriptional activity of DNA-binding transcription factors (TFs) such as NF-κB (11).

Tregs and Th17 cells represent two functionally antagonistic subpopulations of CD4^+^ T cells. Tregs play crucial roles in both moderation of local inflammatory processes and prevention of systemic autoimmunity (12, 13). In solid tumor malignancies the immunosuppressive activity of Tregs can contribute to immune evasion of tumor cells. Indeed, infiltration of Tregs into the tumor is highly correlated with poor prognosis and reduced survival rates (14, 15). On the other hand, even though that the immunological activity of IL-17A and the IL-17A-producing Th17 cells has been associated with immune regulation (16–18), the main immunological activity of Th17 cells is pro-inflammatory and their major physiological role is associated with clearance of extracellular pathogens (19–21). Th17 cells are also associated with various autoimmune diseases, such as psoriasis, rheumatoid arthritis (RA), Crohn’s disease and multiple sclerosis (MS) (22). The pro-inflammatory phenotype of Th17 cells positions them as antagonistic counterparts to tolerogenic Tregs. Intriguingly, the differentiation of CD4^+^ cells into a Th17 subpopulation is very dynamic and includes a shared developmental axis with Tregs (23–25). In this review we highlight the literature that establishes the SRCs as essential regulators of Treg and Th17 cell differentiation, biology, and function. In the context of recent developments in gene editing and small molecule inhibitors (such as SI drugs) and stimulators for SRCs (such as MCB-613), we discuss the potential therapeutic benefit that can arise from targeting the SRCs in Tregs and Th17 cells.

### 1.1 SRC-3 is enriched in Tregs and important for their immune-suppressive function

**Figure 1:**
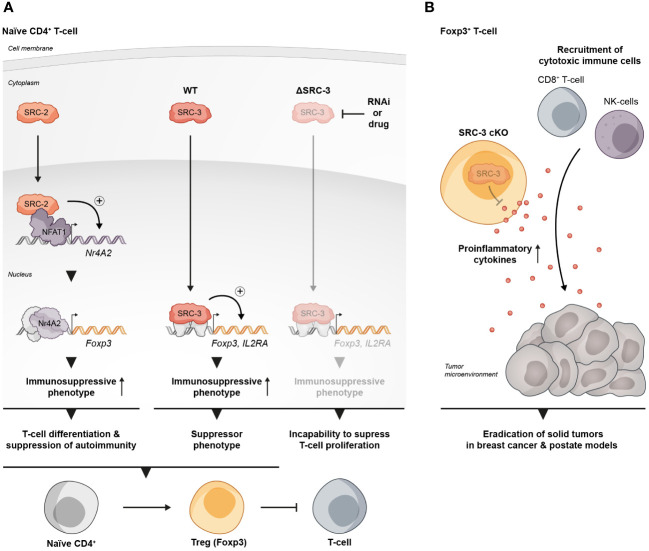
SRCs as regulators of Treg biology. **(A)** SRC-2 and SRC-3 are important regulators of Treg activity: SRC-2 is a transcriptional upregulator of Nr4a2 – a positive TF of Tregs, which makes SRC-2 indispensable for Treg differentiation and suppression of autoimmunity (left). SRC-3 is important for the immunosuppressive activity of Tregs and the inhibition of its activity abolishes the ability of CD4^+^ T cells to acquire a suppressive phenotype (middle and right). **(B)** SRC-3 controls the suppressive activity of Tregs in the TME: Presence of SRC-3 KO FOXP3^+^ T cells reshapes the TME in a way that leads to tumor eradication in breast and prostate cancer mice models.

SRC-3 plays an important role in the regulation of lymphoid cell proliferation, development and function (26–28). It has been demonstrated that SRC-3 deficiency in mice is associated with inflammation (29–31), and that stimulation of SRC-3 with a small molecule stimulator, promotes anti-inflammatory processes such as the establishment of a pro-reparative environment after myocardial infarction (32). These observations suggest a specific role for SRC-3 as a moderator of inflammation. Indeed, interrogation of publicly available databases has revealed that in mice Tregs, SRC-3 expression is significantly higher relative to other lymphocyte subsets, and gene set enrichment analysis (GSEA) has shown that SRC-3 is the second most coexpressed coregulator in cells that express the hallmark transcription factor of Tregs - forkhead box P3 (FOXP3) (33). Similar indications also have been found in human samples showing enriched SRC-3 protein levels in lymphocytes compared to monocytes as well as coexpression of SRC-3 with FOXP3. Moreover, SRC-3 transcript levels were substantially higher in Tregs compared to the bulk population of CD4^+^ T cells, which further bolsters previously mentioned bioinformatic findings. The enriched expression of SRC-3 in Tregs implies its biological significance in this cell type. Indeed, SRC-3 knock down (KD) by RNA interference as well as its pharmacological inhibition in Tregs of human origin brought about a decrease of Treg marker genes, including *FOXP3* and *IL2RA*, at both transcript and protein levels (**Figure 1A**) (33). Further investigation into SRC-3 perturbation has revealed that treatment with an SRC-3 small molecule inhibitor – SI-2 (34) - affects the ability of resting CD4^+^ T cells to adopt a regulatory-like phenotype; generation of induced Tregs (iTregs) in an SRC-3 inhibitor-free environment, resulted in a population of cells with inhibitory activity, as manifested by their ability to suppress the proliferation of conventional CD4^+^ T cells. Conversely, generation of iTregs under pharmacological inhibition of SRC-3 resulted in impaired suppressive activity of these generated iTregs (**Figure 1A**). Furthermore, short-term pharmacological inhibition of SRC-3 in freshly isolated natural Tregs (nTregs) also resulted in a reduction in their suppressive activity (33). Importantly, SI-2 didn’t possess any detectable toxic effects toward CD4^+^ T cells (33), which suggests that the change in their phenotype is attributed to SRC inhibition rather than general cell viability-related effects.

### 1.2 SRC-3 maintains pro-tumorigenic immunosuppression of Tregs

The immunosuppressive activity of Tregs is critical for the maintenance of immune homeostasis, and loss of their canonical suppressive functionality can lead to severe autoimmune disorders (35, 36). In cancer, Tregs can suppress the immune system, which contributes to immune evasion and promotion of tumor development and progression (37). SRC-3 is a pan-cancer oncogene that functions in a cell-autonomous manner within cancer cells, that is strongly associated with multiple malignancies (38–45) and has been particularly well studied in estrogen receptor positive breast cancer (BC) (38, 46–52). Therefore, it is not surprising that small molecules that perturb the activity of SRC-3 were able to significantly suppress the growth of BC cells in numerous *in vitro* and *in vivo* models (29, 53–55). However, intriguingly, it has been demonstrated that the small molecule inhibitor SI-2, can effectively suppresses BC tumor growth in an immune intact mouse at a much lower dose, by a process that is not limited to a direct inhibition of SRC-3 in cancer cells, but instead involves systemic modulation of the immune system and shaping of the composition of infiltrating immune cells in the tumor micro environment (TME) (56). Specifically, the authors demonstrated that treatment of tumor bearing mice with SI-2 is associated with changes in the levels of circulating cytokines in a dose dependent manner; while high-dose treatment with SI-2 was associated with sharp increase of circulating cytokines that leads to systemic toxicity, low-dose treatment led to a moderate increase of cytokines and was associated with an increased infiltration of cytotoxic immune cells and reduced infiltration of Tregs into the TME. Moreover, the authors drew a direct relationship between low-dose SI-2 treatment and elevated levels of cytokines capable of exerting anti-cancer effects, such as IFNγ and CXCL9 – a chemokine that recruits cytotoxic immune cells into the TME. Importantly, low dose treatment with SI-2 of tumor bearing immunodeficient mice failed to suppress tumor growth, which underscores the importance of an intact host immune system toward the totality of the anti-tumor activity that is associated with SRC-3 inhibition. Of note, similar trends were observed in two BC models, when SRC-3 was directly inhibited in tumor cells (shRNA) prior to inoculation; tumor growth was successfully suppressed by SI-2 treatment in a wild type (WT), but not immunodeficient mice. This observation implies that in addition to its role as an inhibitor of the SRC-3 oncogene in cancer cells, SI-2-mediated inhibition of SRC-3 in immune cells plays an essential role in tumor growth inhibition. Overall, this study highlights the centrality of immunomodulatory elements that are associated with SI-2-mediated anti-cancer activity.

A more direct piece of evidence that SRC-3 perturbation in a specific type of immune cells contributes to tumor eradication was revealed in another study where it has been shown that a conditional KO (cKO) of SRC-3 in Tregs results in complete tumor clearance in an *in vivo* BC model (57). Using a syngeneic BC mouse cancer cell line in conjunction with the SRC-3 Treg cKO mice, the authors demonstrated that tumor eradication can be achieved when SRC-3 KO takes place in Tregs following tamoxifen induction of Cre-recombinase. SRC-3 KO in Tregs results in increased infiltration of cytotoxic immune cells into the tumor and elevated levels of IFNγ and CXCL9 in the TME. These observations strikingly recapitulate the effects of pharmacological inhibition of SRC-3 (56) and provide mechanistic insight into the anti-tumor phenotype of the SRC-3 KO Tregs. In the light of these data, the authors suggest a model where tumor-specific accumulation of the edited Tregs that secrete IFNγ, results in elevated levels of IFNγ in the TME. In the tumor, IFNγ stimulates the production of CXCL9 by tumor and stromal cells, resulting in increased infiltration of CXCR3^+^ (CXCL9 receptor) cytotoxic immune cells (such as CD8^+^ T cells and Natural Killer cells) into the tumor that promotes its eradication (**Figure 1B**). Importantly, blockade of IFNγ through anti-IFNγ antibody (Ab) treatment, abolished the anti-tumor therapeutic effect of SRC-3 KO Tregs, which further solidifies the centrality of the IFNγ/CXCL9 axis to SRC-3 KO Treg-mediated tumor clearance. Of note, re-inoculation of cancer cells into the breast cancer-cured mice did not result in a newly developed tumor, demonstrating a long-term immunization-like effect of the SRC-3 KO Tregs. The authors also showed that SRC-3 KO Tregs can be harvested from tamoxifen-treated genetically engineered SRC-3^f/f^:FOXP3^Cre-ERT2/+^ mice and then injected into tumor bearing mice, resulting in tumor eradication, thus demonstrating the translational therapeutic potential of this technology. Notably, the Treg cell-specific SRC-3 KO that leads to permanent eradication of solid tumors was not associated with any observed pathological phenotype, including autoimmunity or reproductive defects (56, 57). Complete tumor eradication that takes place in the absence of any autoimmune side effect is a striking outcome of this therapy. It can be partially explained by the concentration of SRC3 KO-Tregs in the TME, where they secrete IFNγ and activate the IFNγ/CXCL9 axis. Local activation of the IFNγ/CXCL9 axis, with no detectable increase in IFNγ within the lymphatic system, results in an immunologically hot (inflamed) tumor, but with minimal impact outside of the TME (58). Also, the fact that the three SRC-family members can compensate for one another, sets the foundation for reasonable speculation that SRC-1/2 compensate for SRC-3 activity in SRC3 KO-Tregs outside of the TME to support their immune-regulatory function and maintain homeostasis. However, there is yet more light to be shed on the exact mechanism by which SRC-3 KO shapes the phenotype of the SRC-3 KO-Tregs, that drives their trafficking into the tumor, and the possible role that the compensatory mechanisms between each SRC plays in the maintenance of Treg-mediated immune homeostasis.

### 1.3 SRC-2 is critical for CD4^+^ T cells activation and differentiation into FOXP3^+^ expressing Tregs

The role of SRC-2 as a coregulator of metabolic processes is very well established (59–62). In CD4^+^ T cells SRC-2 controls the upregulation of Slc7a5 (63) - an amino acid transporter that is crucial for amino acid supply from the extracellular environment into stimulated CD4^+^ T cells to fulfill the requirement of these cells for cytokine production and proliferation. CD4^+^ T cell-specific deficiency of SRC-2 results in resistance to autoimmune disorders and an impaired immune response, as has been demonstrated using experimental autoimmune encephalomyelitis (EAE) and colitis mice models (63). Importantly, forced expression of Slc7a5 in SRC-2 deficient CD4^+^ T cells restored the ability to induce EAE and rescued the proliferation defect of these cells, indicating that SRC-2 impacts CD4^+^ T cells in a specific manner through the regulation of Slc7a5 expression. Interestingly, in Tregs, SRC-2 is not required for either proliferation or survival and deficiency of SRC-2 does not affect thymic development of natural Tregs (nTregs), as demonstrated by equal FOXP3 expression in CD4^+^ thymocytes regardless of whether the origin of these cells is FOXP3^YFP-Cre^ or SRC2^fl/fl^:FOXP3^YFP-Cre^ mice (64). However, SRC-2 is essential for the ability of naïve CD4^+^ T cells to adopt a Treg phenotype *in vitro* in the presence of transforming growth factor beta (TGFβ) (64). Moreover, when FOXP3^-^ CD4^+^ T cells were adoptively transferred into Rag^-/-^ recipient mice which lack the ability to produce mature T cells, they were able to establish stable FOXP3 expression, but when SRC-2 was cKO in a FOXP3-dependent manner, these cells fail to express FOXP3 (64). This indicates that SRC-2 is indispensable for the ability of CD4^+^ T cells to differentiate into Tregs *in vivo* with the implication that at a systemic level, mice with SRC-2 deficient Tregs have significantly higher concentrations of pro-inflammatory cytokines compared to their WT counterparts. Accordingly, these mice are at a much higher risk for autoimmune disorders as has been demonstrated by their inability to be protected from EAE. Supportive of these observations, aged SRC2^fl/fl^:FOXP3^YFP-Cre^ mice spontaneously developed autoimmune-like phenotypes, manifested by splenomegaly, reduced FOXP3 expression and limited immuno-suppressive activity in older animals (64). Eventually these phenotypic hallmarks of unrestrained immunity bring about increased numbers of pro-inflammatory T cells in the lungs, with subsequent tissue damage. Mechanistically, SRC-2 cKO in Tregs leads to the downregulation of four TFs - Myb, Irf4, Foxo1, and Nr4a2 – which all positively contribute to Treg differentiation. Additionally, ChIP-qPCR and immunoprecipitation assays revealed that SRC-2 is recruited by the TF NFAT1 to the Nr4a2 promoter (**Figure 1A**). Moreover, deletion of NFAT1/SRC-2 DNA binding sites significantly reduced Nr4a2 mRNA and protein expression. Furthermore, forced expression of Nr4a2 in SRC-2 deficient Tregs resulted in restored FOXP3 expression and the ability of SRC2^fl/fl^:FOXP3^YFP-Cre^ cells to differentiate into Tregs. Collectively, these observations underscore the crucial role of SRC-2 in the transcriptional upregulation of Nr4a2, which solidifies its significance for proper differentiation and function of Tregs.

### 1.4 SRC-1 and SRC-3 are critical regulators of Th17 cell differentiation

Th17 cells represent a pro-inflammatory subtype of CD4^+^ T cells, that are critically important for the clearance of extracellular pathogens but also are often associated with autoimmune disorders (21). Retinoic acid-related orphan receptor (ROR)γt is a hallmark TF expressed in Th17 cells, since it regulates the production of the signature cytokine produced by these cells - IL17A (65, 66). Though Th17 cells and Tregs both originate from naïve CD4^+^ T cells, once they commit to their specific phenotype, they become reciprocally inhibitory toward each other’s function and differentiation. Treg *vs* Th17 cell fate decision of CD4^+^ T cells at their early developmental stages might partially rely on TGFβ signaling, and fine regulation is required at these stages for the stable lineage commitment of both cell types (67–69). TGFβ-driven determination of Treg *vs* Th17 cell fate ultimately depends on the cytokine environment in which the TGFβ signaling takes place, since the cytokine milieu regulates the equilibrium of the competitive interplay between FOXP3 and ROR-family TFs in developing CD4^+^ T cells (70). Furthermore, in certain biological contexts, Tregs have the potential to undergo redifferentiation and transform into effector Th17-like cells. This transformation is marked by reduced expression of FOXP3 and the ability to produce IL17A (2, 25).

**Figure 2:**
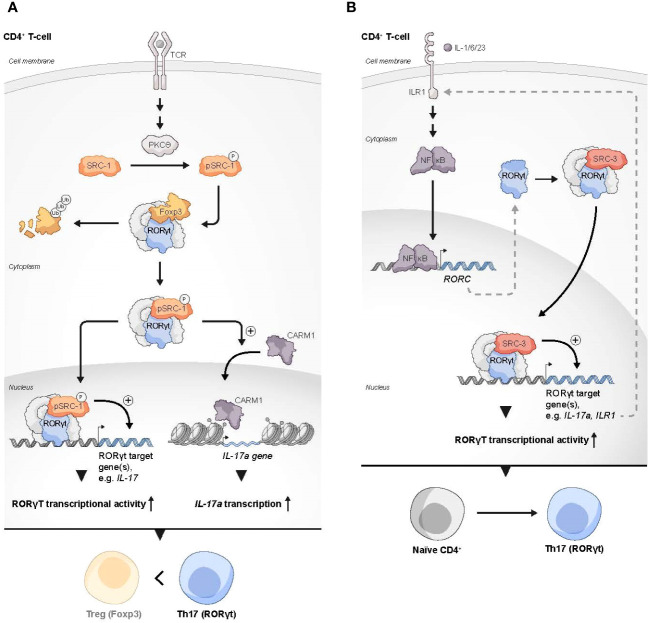
SRCs as regulators of Th17 cells. **(A)** Two possible mechanisms by which SRC-1 contributes to Th17 differentiation and phenotype dominance over Tregs: 1) PKCθ-driven phosphorylation of SRC-1 enhances its interaction with RORγt, which results in dominance of RORγt over FOXP3 transcriptional activity. 2) The SRC-1-RORγt complex promotes the recruitment of CARM1 to the IL-17 locus to generate a permissive chromatin structure that leads to enhanced transcription of the IL17A gene. **(B)** SRC-3 regulates the expression of genes in Th17 cells through the IL-1 mediated signaling axis: Under stimulation with IL-1/6/23, a RORγt-SRC-3 complex is recruited to the promoters of the IL17A and IL17R1 genes to induce their expression, resulting in the polarization of naïve CD4^+^ T cells into the Th17 cell lineage.

RORγt uses its activation function (AF)2 domain to recruit SRC-1 and SRC-2 through the interaction with their LXXLL motifs (71). In thymocytes, RORγt recruitment of SRCs is necessary for a robust RORγt-mediated transcriptional activity and thymocyte survival, while during later stages of T cell development, the interaction of SRC-1 with RORγt is critically important for the establishment of the Th17 phenotype (71). The mechanism that underlies RORγt-SRC-1 transcriptional cooperation involves protein kinase C (PKC)-θ mediated phosphorylation of SRC-1 conserved serines 1271 and 1272 (72). Phosphorylated SRC-1 has an enhanced engagement with RORγt. As a result of the strong engagement of RORγt with the phosphorylated SRC-1, FOXP3 that binds directly to RORγt and antagonizes its transcriptional activity (70, 73) is displaced from its complex with RORγt and subjected to proteosomal degradation, while an SRC-1-bound RORγt acquires a higher DNA-binding capability and transcriptional activity (72). Moreover, the SRC-1-RORγt complex promotes the recruitment of the CARM1 methyltransferase to the *il17* locus to modify its chromatin methylation pattern and generate a permissive chromatin structure that leads to amplified IL-17a transcription (72). This unveils the two roles that SRC-1 has in promoting the differentiation of Th17 cells, either by direct up-regulation of RORγt activity and/or recruitment of CARM1 to key loci of genes that drive the Th17 cell phenotype. Collectively, SRC-1 activity skews the balance between FOXP3 and RORγt programs towards the latter, which leads to a phenotypic dominance of Th17 cells and a simultaneous decrease in the Treg phenotype (**Figure 2A**).

Like SRC-1, SRC-3 also plays an essential role in RORγt-driven Th17 cell differentiation. Through a physical interaction with RORγt and under specific proinflammatory stimulation with IL-1/6/23, SRC-3 is recruited to the *il17* and *il1r1* loci to polarize naïve CD4^+^ T cells into the Th17 cell lineage (74) (**Figure 2B**). SRC-3-mediated commitment of naïve CD4^+^ T cells to the Th17 cell lineage manifests in an upregulation of Th17 cell signature genes and an increased number of IL-17 producing cells among WT CD4^+^ T cells as compared to SRC-3-KO CD4^+^ T cells. Reduced binding of the acetyltransferase p300 to the *il17* and *il1r1* loci, that is associated with SRC-3 deficiency, suggests that an RORγt-mediated recruitment of SRC-3 brings about a recruitment of p300 to establish an open chromatin structure that facilitates the transcription of Th17 signature genes. Importantly, a K313R mutation of RORγt that specifically disrupts the ability of RORγt to interact with SRC-3 but not SRC-1, impairs healthy differentiation and development of Th17, but not thymocytes (75). Intriguingly, under TGFβ/IL-6 stimulation, SRC-3 deficient naïve CD4^+^ T cells successfully adopt the Th17 cell phenotype (74). This implies two different pathways for Th17 cell differentiation, and that the SRC-3-driven one utilizes an IL-1/IL1R1 signaling axis. However, further investigation is required to shed more light on the details of the mechanistic differences between these pathways. As previously mentioned, exposure of naive human CD4^+^ T cells to a pharmacological inhibitor of SRC-3 affects the ability of these cells to acquire a suppressive phenotype under Treg-inducing conditions *ex vivo* (33). Nonetheless, genetic deficiency of SRC-3 in a mouse model did not appear to impair ‘naïve CD4^+^ T cells to Tregs’ polarization (74), This apparent discrepancy might reflect differences in the manner that the Treg phenotype is acquired (*e.g.*, *ex vivo* induction versus *in vivo* natural polarization) as well as variations between the model organisms. However, as opposed to the neutral effect of SRC-3 deficiency in mice toward Treg polarization, the increase in the Treg-like phenotype that is associated with SRC-1 deficiency (72), suggests a potentially different nature of interactions of these two coactivators with the RORγt-FOXP3 complex.

## 2 Summary and future perspectives

Since the discovery and cloning of the first SRC, SRC-1, almost 30 years ago (6), these coactivators have been established as critical regulators of gene expression with broad range of impact on human physiology and pathology. Specifically in immunology, the SRCs have diverse biological functionalities that include interactions with major immune system TFs such as NF-κB and RORγt, involvement in immune cell fate determination and development as well as control of their proliferation (11). In this review, we have highlighted the roles of the SRCs in the biology of two, functionally antagonistic, subtypes of CD4^+^ T cells – Tregs and Th17 cells; SRC-3 is one of the most highly expressed coactivators in Tregs and is important for their suppressive function. Indeed, the importance of SRC-3 to Treg biology is reflected in the anti-tumor phenotype of SRC-3 deficient Tregs, that when recruited to the TME exert a pro-inflammatory phenotype which drives the infiltration of immune effector cells into the tumor and eventually brings about tumor eradication. SRC-2 is indispensable for the development and proper function of Tregs, since it regulates the expression of a panel of TFs that are important for Treg differentiation, including Nr4a2, that directly targets FOXP3 and regulates Tregs suppression activity (76).

All SRCs can physically interact with the major TF of Th17 cells, RORγt, through their conserved NR binding LXXLL motif, pointing to the significant role that the SRCs play in Th17 cell biology. Indeed, it has been shown that PKC-θ-mediated phosphorylation of SRC-1 enhances its physical interaction with RORγt and drives the establishment of Th17 cell phenotype, rather than the Treg phenotype in naïve CD4^+^ T cells. Under inflammatory conditions, SRC-3 interaction with RORγt promotes pathogenic inflammation through the IL-1/ILR1 signaling axis. The positive contribution of the SRCs to the establishment of Th17 cell phenotype has physiological implications as reflected in the resistance to autoimmunity that is associated with SRC-1 and SRC-3 deficiency in the EAE mice model (72, 74).

The SRCs have historically been considered ‘undruggable’ proteins, primarily due to their lack of high-affinity ligand binding pockets (7, 77). However, recent success in developing small molecule inhibitors and stimulators counter this view and show that it is possible to manipulate the activity of the SRCs (29, 34), presenting an opportunity for new therapeutic venues. Unsurprisingly, since the SRCs are very well established oncoproteins, their conception as pharmacological targets was first directed toward malignant diseases. However, recent accumulation of evidence that established the SRCs as important coregulators in the biology and function of Th17 cells and Tregs, implies their manipulation in these immune cells in autoimmune disorders and cancer as an exciting new immunotherapeutic area for development. Indeed, it has been shown that a systemic treatment with an SRC-3 inhibitor significantly suppressed BC progression in a syngeneic mouse model, not only in part due to a direct inhibition of SRC-3 in cancer cells, but primarily due to the immunological boost induced by the inhibitor (56). A recent study has shown that a cKO of SRC-3 in Tregs results in tumor eradication in BC and prostate cancer mouse models (57), inferring a direct impact of SRC-3 on cancer immunology. Moreover, the demonstration that *ex vivo* edited SRC-3 KO Tregs can be adaptively transferred to effect tumor eradication, provides the impetus for the development of an SRC-3 KO gene edited Treg-based cell therapy for solid tumors.

These recent studies unveil the immense therapeutic potential of manipulating the activity of SRCs in immune cells (**Table 1**), and future studies should shed more light on the translational potential of this approach by leveraging the discovery of small molecules that selectively target the SRCs as well as powerful CRISPR-Cas based gene editing tools (78). The concept of inhibiting Th17 cell activity in an autoimmune disease through the use of small molecules that target the RORs has been recently introduced (79, 80). The key role of SRCs in Th17 cell biology potentiates pharmacological targeting of these proteins as an alternative approach to treat this class of disorders. However, further research is necessary to validate these approaches.

**Table 1:** Changes in Tregs and Th17 phenotypes associated with manipulations of SRC activities.

SRC protein	Manipulation	Observed phenotype	Reference
SRC-1	Overexpression	Enhanced differentiation of naïve CD4^+^ T cells into IL-17 producing cells *in vitro*.	(72)
	KD	Reduce production of IL-17 by CD4^+^ T cells *in vitro*.	
	KO	Decrease percentage of IL-17 producing cells and increased percentage of FOXP3-expressing cells among general lymphocyte population.	
	KO	Immunosuppressive phenotype dominance that is exemplified by resistance of SRC-1^-/-^ mice to the development of EAE.	
SRC-2	KO	cKO in FOXP3^+^ cells results in impaired ability of naïve CD4^+^ T cells to differentiate into iTregs *in vitro* under priming conditions with TGFβ.	(64)
	KO	cKO in FOXP3^+^ cells impairs the ability of these cells to acquire Treg phenotype *in vivo*.	
SRC-3	none	Enriched in Tregs.	(33)
	pharmacological inhibition	Failure of resting CD4^+^ T cells to adapt Treg phenotype under inducible Treg condition *in vitro.*	
	pharmacological inhibition	Inhibition of tumor growth in BC mouse models after systemic treatment with SI-2, which is associated with a decrease of FOXP3^+^ cells and increase of cytotoxic immune cells (CD8^+^ T cells, NK cells) and proinflammatory cytokines in the TME.	(56)
	KO	cKO in FOXP3^+^ cells *in vivo* induces elevated levels of proinflammatory cytokines and increase of cytotoxic immune cells (CD8^+^ T cells, NK cells) in the TME and eradication of solid tumors in mice (BC and prostate cancer models).	(57)

## Author contributions

YG: Conceptualization, Visualization, Writing – original draft, Writing – review & editing. OS: Writing – original draft, Writing – review & editing. SH: Writing – original draft, Writing – review & editing. DML: Writing – original draft, Writing – review & editing. BWO: Writing – original draft, Writing – review & editing.

## References

[1] Zhu J Paul WE. CD4 T cells: fates, functions, and faults. Blood. (2008) 112:1557–69. doi: 10.1182/blood-2008-05-078154 PMC2518872 18725574

[2] Zhou L Chong MM Littman DR. Plasticity of CD4+ T cell lineage differentiation. Immunity. (2009) 30:646–55. doi: 10.1016/j.immuni.2009.05.001 19464987

[3] Loo TT Gao Y Lazarevic V. Transcriptional regulation of CD4+ TH cells that mediate tissue inflammation. J Leukoc Biol. (2018) 104:1069–85. doi: 10.1002/JLB.1RI0418-152RR PMC6662913 30145844

[4] De Bosscher K Desmet SJ Clarisse D Estébanez-Perpiña E Brunsveld L. Nuclear receptor crosstalk—defining the mechanisms for therapeutic innovation. Nat Rev Endocrinol. (2020) 16:363–77. doi: 10.1038/s41574-020-0349-5 32303708

[5] Weikum ER Liu X Ortlund EA. The nuclear receptor superfamily: A structural perspective. Protein Sci. (2018) 27:1876–92. doi: 10.1002/pro.3496 PMC6201731 30109749

[6] Onate SA Tsai SY Tsai M-J O'Malley BW. Sequence and characterization of a coactivator for the steroid hormone receptor superfamily. Science. (1995) 270:1354–7. doi: 10.1126/science.270.5240.1354 7481822

[7] Lonard DM O'malley BW. Nuclear receptor coregulators: modulators of pathology and therapeutic targets. Nat Rev Endocrinol. (2012) 8:598–604. doi: 10.1038/nrendo.2012.100 22733267 PMC3564250

[8] Dasgupta S Lonard DM O'Malley BW. Nuclear receptor coactivators: master regulators of human health and disease. Annu Rev Med. (2014) 65:279–92. doi: 10.1146/annurev-med-051812-145316 PMC4327818 24111892

[9] York B O'Malley BW. Steroid receptor coactivator (SRC) family: masters of systems biology. J Biol Chem. (2010) 285:38743–50. doi: 10.1074/jbc.R110.193367 PMC2998129 20956538

[10] Mullany LK Lonard DM O’Malley BW. Wound healing-related functions of the p160 steroid receptor coactivator family. Endocrinology. (2020) 162. doi: 10.1210/endocr/bqaa232 PMC7814297 33340403

[11] Gilad Y Lonard DM O’Malley BW. Steroid receptor coactivators–their role in immunity. Front Immunol. (2022) 13:1079011. doi: 10.3389/fimmu.2022.1079011 36582250 PMC9793089

[12] Plitas G Rudensky AY. Regulatory T cells: differentiation and function. Cancer Immunol Res. (2016) 4:721–5. doi: 10.1158/2326-6066.CIR-16-0193 PMC5026325 27590281

[13] Alroqi FJ Chatila TA. T regulatory cell biology in health and disease. Curr Allergy Asthma Rep. (2016) 16:1–8. doi: 10.1007/s11882-016-0606-9 26922942 PMC5218767

[14] Tanchot C Terme M Pere H Tran T Benhamouda N Strioga M. Tumor-infiltrating regulatory T cells: phenotype, role, mechanism of expansion *in situ* and clinical significance. Cancer Microenviron. (2013) 6:147–57. doi: 10.1007/s12307-012-0122-y PMC3717062 23104434

[15] Tanaka A Sakaguchi S. Regulatory T cells in cancer immunotherapy. Cell Res. (2017) 27:109–18. doi: 10.1038/cr.2016.151 PMC5223231 27995907

[16] Xu H Agalioti T Zhao J Steglich B Wahib R Vesely MCA. The induction and function of the anti-inflammatory fate of T(H)17 cells. Nat Commun. (2020) 11:3334. doi: 10.1038/s41467-020-17097-5 32620760 PMC7335205

[17] Brockmann L Tran A Huang Y Edwards M Ronda C Wang HH. Intestinal microbiota-specific Th17 cells possess regulatory properties and suppress effector T cells via c-MAF and IL-10. Immunity. (2023) 56:2719–2735.e7. doi: 10.1016/j.immuni.2023.11.003 38039966 PMC10964950

[18] O'Connor W Jr. Kamanaka M Booth CJ Town T Nakae S Iwakura Y. A protective function for interleukin 17A in T cell-mediated intestinal inflammation. Nat Immunol. (2009) 10:603–9. doi: 10.1038/ni.1736 PMC2709990 19448631

[19] Harrington LE Hatton RD Mangan PR Turner H Murphy TL Murphy KM. Interleukin 17–producing CD4+ effector T cells develop via a lineage distinct from the T helper type 1 and 2 lineages. Nat Immunol. (2005) 6:1123–32. doi: 10.1038/ni1254 16200070

[20] Park H Li Z Yang XO Chang SH Nurieva R Wang Y-H. A distinct lineage of CD4 T cells regulates tissue inflammation by producing interleukin 17. Nat Immunol. (2005) 6:1133–41. doi: 10.1038/ni1261 PMC1618871 16200068

[21] Mills KHG. IL-17 and IL-17-producing cells in protection versus pathology. Nat Rev Immunol. (2023) 23:38–54. doi: 10.1038/s41577-022-00746-9 35790881 PMC9255545

[22] Stockinger B Omenetti S. The dichotomous nature of T helper 17 cells. Nat Rev Immunol. (2017) 17:535–44. doi: 10.1038/nri.2017.50 28555673

[23] Gagliani N Vesely Amezcua MC Iseppon A Brockmann L Xu H Palm NW. Th17 cells transdifferentiate into regulatory T cells during resolution of inflammation. Nature. (2015) 523:221–5. doi: 10.1038/nature14452 PMC4498984 25924064

[24] Yang XO Nurieva R Martinez GJ Kang HS Chung Y Pappu BP. Molecular antagonism and plasticity of regulatory and inflammatory T cell programs. Immunity. (2008) 29:44–56. doi: 10.1016/j.immuni.2008.05.007 18585065 PMC2630532

[25] Weaver CT Hatton RD. Interplay between the TH17 and TReg cell lineages: a (co-)evolutionary perspective. Nat Rev Immunol. (2009) 9:883–9. doi: 10.1038/nri2660 19935807

[26] Coste A Antal MC Chan S Kastner P Mark M O'Malley BW. Absence of the steroid receptor coactivator-3 induces B-cell lymphoma. EMBO J. (2006) 25:2453–64. doi: 10.1038/sj.emboj.7601106 PMC1478181 16675958

[27] Hu M Zeng H Chen S Xu Y Wang S Tang Y. SRC-3 is involved in maintaining hematopoietic stem cell quiescence by regulation of mitochondrial metabolism in mice. Blood. (2018) 132:911–23. doi: 10.1182/blood-2018-02-831669 29959189

[28] Hu M Lu Y Qi Y Zhang Z Wang S Xu Y. SRC-3 functions as a coactivator of T-bet by regulating the maturation and antitumor activity of natural killer cells. Cancer Immunol Res. (2020) 8:1150–62. doi: 10.1158/2326-6066.CIR-20-0181 32561537

[29] Wang L Yu Y Chow DC Yan F Hsu CC Stossi F. Characterization of a steroid receptor coactivator small molecule stimulator that overstimulates cancer cells and leads to cell stress and death. Cancer Cell. (2015) 28:240–52. doi: 10.1016/j.ccell.2015.07.005 PMC4536575 26267537

[30] Yu C York B Wang S Feng Q Xu J O'Malley BW. An essential function of the SRC-3 coactivator in suppression of cytokine mRNA translation and inflammatory response. Mol Cell. (2007) 25:765–78. doi: 10.1016/j.molcel.2007.01.025 PMC1864954 17349961

[31] Chen Q Chen T Xu Y Zhu J Jiang Y Zhao Y. Steroid receptor coactivator 3 is required for clearing bacteria and repressing inflammatory response in Escherichia coli-induced septic peritonitis. J Immunol. (2010) 185:5444–52. doi: 10.4049/jimmunol.0903802 PMC3640995 20881187

[32] Mullany LK Rohira AD Leach JP Kim JH Monroe TO Ortiz AR. A steroid receptor coactivator stimulator (MCB-613) attenuates adverse remodeling after myocardial infarction. Proc Natl Acad Sci U.S.A. (2020) 117:31353–64. doi: 10.1073/pnas.2011614117 PMC7733826 33229578

[33] Nikolai BC Jain P Cardenas DL York B Feng Q McKenna NJ. Steroid receptor coactivator 3 (SRC-3/AIB1) is enriched and functional in mouse and human Tregs. Sci Rep. (2021) 11:3441. doi: 10.1038/s41598-021-82945-3 33564037 PMC7873281

[34] Song X Chen J Zhao M Zhang C Yu Y Lonard DM. Development of potent small-molecule inhibitors to drug the undruggable steroid receptor coactivator-3. Proc Natl Acad Sci U.S.A. (2016) 113:4970–5. doi: 10.1073/pnas.1604274113 PMC4983835 27084884

[35] Rajendeeran A Tenbrock K. Regulatory T cell function in autoimmune disease. J Transl Autoimmun. (2021) 4:100130. doi: 10.1016/j.jtauto.2021.100130 35005594 PMC8716637

[36] Dominguez-Villar M Hafler DA. Regulatory T cells in autoimmune disease. Nat Immunol. (2018) 19:665–73. doi: 10.1038/s41590-018-0120-4 PMC7882196 29925983

[37] Togashi Y Shitara K Nishikawa H. Regulatory T cells in cancer immunosuppression — implications for anticancer therapy. Nat Rev Clin Oncol. (2019) 16:356–71. doi: 10.1038/s41571-019-0175-7 30705439

[38] Anzick SL Kononen J Walker RL Azorsa DO Tanner MM Guan XY. AIB1, a steroid receptor coactivator amplified in breast and ovarian cancer. Science. (1997) 277:965–8. doi: 10.1126/science.277.5328.965 9252329

[39] Sakaguchi H Fujimoto J Sun WS Tamaya T. Clinical implications of steroid receptor coactivator (SRC)-3 in uterine endometrial cancers. J Steroid Biochem Mol Biol. (2007) 104:237–40. doi: 10.1016/j.jsbmb.2007.03.007 17532621

[40] Balmer NN Richer JK Spoelstra NS Torkko KC Lyle PL Singh M. Steroid receptor coactivator AIB1 in endometrial carcinoma, hyperplasia and normal endometrium: Correlation with clinicopathologic parameters and biomarkers. Mod Pathol. (2006) 19:1593–605. doi: 10.1038/modpathol.3800696 16980945

[41] Ghadimi BM Schröck E Walker RL Wangsa D Jauho A Meltzer PS. Specific chromosomal aberrations and amplification of the AIB1 nuclear receptor coactivator gene in pancreatic carcinomas. Am J Pathol. (1999) 154:525–36. doi: 10.1016/S0002-9440(10)65298-4 PMC1850008 10027410

[42] Yoshida H Liu J Samuel S Cheng W Rosen D Naora H. Steroid receptor coactivator-3, a homolog of Taiman that controls cell migration in the Drosophila ovary, regulates migration of human ovarian cancer cells. Mol Cell Endocrinol. (2005) 245:77–85. doi: 10.1016/j.mce.2005.10.008 16298470

[43] Henke RT Haddad BR Kim SE Rone JD Mani A Jessup JM. Overexpression of the nuclear receptor coactivator AIB1 (SRC-3) during progression of pancreatic adenocarcinoma. Clin Cancer Res. (2004) 10:6134–42. doi: 10.1158/1078-0432.CCR-04-0561 15448000

[44] Mo P Zhou Q Guan L Wang Y Wang W Miao M. Amplified in breast cancer 1 promotes colorectal cancer progression through enhancing notch signaling. Oncogene. (2015) 34:3935–45. doi: 10.1038/onc.2014.324 PMC4377317 25263446

[45] He LR Zhao HY Li BK Zhang LJ Liu MZ Kung HF. Overexpression of AIB1 negatively affects survival of surgically resected non-small-cell lung cancer patients. Ann Oncol. (2010) 21:1675–81. doi: 10.1093/annonc/mdp592 20064830

[46] Bautista S Vallès H Walker RL Anzick S Zeillinger R Meltzer P. In breast cancer, amplification of the steroid receptor coactivator gene AIB1 is correlated with estrogen and progesterone receptor positivity. Clin Cancer Res. (1998) 4:2925–9. 9865902

[47] Bouras T Southey MC Venter DJ. Overexpression of the steroid receptor coactivator AIB1 in breast cancer correlates with the absence of estrogen and progesterone receptors and positivity for p53 and HER2/neu. Cancer Res. (2001) 61:903–7. 11221879

[48] Zhao C Yasui K Lee CJ Kurioka H Hosokawa Y Oka T. Elevated expression levels of NCOA3, TOP1, and TFAP2C in breast tumors as predictors of poor prognosis. Cancer. (2003) 98:18–23. doi: 10.1002/cncr.11482 12833450

[49] Osborne CK Bardou V Hopp TA Chamness GC Hilsenbeck SG Fuqua SA. Role of the estrogen receptor coactivator AIB1 (SRC-3) and HER-2/neu in tamoxifen resistance in breast cancer. J Natl Cancer Inst. (2003) 95:353–61. doi: 10.1093/jnci/95.5.353 12618500

[50] Kuang SQ Liao L Zhang H Lee AV O'Malley BW Xu J. AIB1/SRC-3 deficiency affects insulin-like growth factor I signaling pathway and suppresses v-Ha-ras-induced breast cancer initiation and progression in mice. Cancer Res. (2004) 64:1875–85. doi: 10.1158/0008-5472.CAN-03-3745 14996752

[51] Fereshteh MP Tilli MT Kim SE Xu JBW Wellstein A. The nuclear receptor coactivator amplified in breast cancer-1 is required for Neu (ErbB2/HER2) activation, signaling, and mammary tumorigenesis in mice. Cancer Res. (2008) 68:3697–706. doi: 10.1158/0008-5472.CAN-07-6702 PMC3641830 18483252

[52] Zhao Y Laws MJ Guillen VS Ziegler Y Min J Sharma A. Structurally novel antiestrogens elicit differential responses from constitutively active mutant estrogen receptors in breast cancer cells and tumors. Cancer Res. (2017) 77:5602–13. doi: 10.1158/0008-5472.CAN-17-1265 PMC5645250 28904064

[53] Gilad Y Eliaz Y Yu Y Dean AM Han SJ Qin L. A genome-scale CRISPR Cas9 dropout screen identifies synthetically lethal targets in SRC-3 inhibited cancer cells. Commun Biol. (2021) 4:399. doi: 10.1038/s42003-021-01929-1 33767353 PMC7994904

[54] Qin L Chen J Lu D Jain P Yu Y Cardenas D. Development of improved SRC-3 inhibitors as breast cancer therapeutic agents. Endocr Relat Cancer. (2021) 28:657–70. doi: 10.1530/ERC-20-0402 PMC8404148 34310341

[55] Gilad Y Eliaz Y Yu Y Han SJ O’Malley BW Lonard DM. Drug-induced PD-L1 expression and cell stress response in breast cancer cells can be balanced by drug combination. Sci Rep. (2019) 9:15099. doi: 10.1038/s41598-019-51537-7 31641154 PMC6805932

[56] Han SJ Sung N Wang J O'Malley BW Lonard DM. Steroid receptor coactivator-3 inhibition generates breast cancer antitumor immune microenvironment. Breast Cancer Res. (2022) 24:73. doi: 10.1186/s13058-022-01568-2 36316775 PMC9620627

[57] Han SJ Jain P Gilad Y Xia Y Sung N Park MJ. Steroid receptor coactivator 3 is a key modulator of regulatory T cell–mediated tumor evasion. Proc Natl Acad Sci U.S.A. (2023) 120:e2221707120. doi: 10.1073/pnas.2221707120 37253006 PMC10266015

[58] Nagarsheth N Wicha MS Zou W. Chemokines in the cancer microenvironment and their relevance in cancer immunotherapy. Nat Rev Immunol. (2017) 17:559–72. doi: 10.1038/nri.2017.49 PMC5731833 28555670

[59] Dasgupta S Putluri N Long W Zhang B Wang J Kaushik AK. Coactivator SRC-2-dependent metabolic reprogramming mediates prostate cancer survival and metastasis. J Clin Invest. (2015) 125:1174–88. doi: 10.1172/JCI76029 PMC4362260 25664849

[60] Stashi E Lanz RB Mao J Michailidis G Zhu B Kettner NM. SRC-2 is an essential coactivator for orchestrating metabolism and circadian rhythm. Cell Rep. (2014) 6:633–45. doi: 10.1016/j.celrep.2014.01.027 PMC4096300 24529706

[61] Stashi E York B O'Malley BW. Steroid receptor coactivators: servants and masters for control of systems metabolism. Trends Endocrinol Metab. (2014) 25:337–47. doi: 10.1016/j.tem.2014.05.004 PMC4108168 24953190

[62] O'Malley BW. SRC-2 Coactivator: a role in human metabolic evolution and disease. Mol Med. (2020) 26:45. doi: 10.1186/s10020-020-00168-0 32410572 PMC7227291

[63] Zhang W Cao X Zhong X Wu H Shi Y Feng M. SRC2 controls CD4+ T cell activation via stimulating c-Myc-mediated upregulation of amino acid transporter Slc7a5. Proc Natl Acad Sci U.S.A. (2023) 120:e2221352120. doi: 10.1073/pnas.2221352120 37094160 PMC10160970

[64] Zhang W Cao X Zhong X Wu H Feng M Gwack Y. Steroid nuclear receptor coactivator 2 controls immune tolerance by promoting induced T(reg) differentiation via up-regulating Nr4a2. Sci Adv. (2022) 8:eabn7662. doi: 10.1126/sciadv.abn7662 35704583 PMC9200286

[65] Ivanov II McKenzie BS Zhou L Tadokoro CE Lepelley A Lafaille JJ. The orphan nuclear receptor RORgammat directs the differentiation program of proinflammatory IL-17+ T helper cells. Cell. (2006) 126:1121–33. doi: 10.1016/j.cell.2006.07.035 16990136

[66] Yang XO Pappu BP Nurieva R Akimzhanov A Kang HS Chung Y. T helper 17 lineage differentiation is programmed by orphan nuclear receptors ROR alpha and ROR gamma. Immunity. (2008) 28:29–39. doi: 10.1016/j.immuni.2007.11.016 18164222 PMC2587175

[67] Wang J Zhao X Wan YY. Intricacies of TGF-β signaling in Treg and Th17 cell biology. Cell Mol Immunol. (2023) 20:1002–22. doi: 10.1038/s41423-023-01036-7 PMC10468540 37217798

[68] Chen W. TGF-β Regulation of T cells. Annu Rev Immunol. (2023) 41:483–512. doi: 10.1146/annurev-immunol-101921-045939 36750317 PMC12453633

[69] Zhang S. The role of transforming growth factor β in T helper 17 differentiation. Immunology. (2018) 155:24–35. doi: 10.1111/imm.12938 29682722 PMC6099164

[70] Zhou L Lopes JE Chong MMW Ivanov II Min R Victora GD. TGF-β-induced Foxp3 inhibits TH17 cell differentiation by antagonizing RORγt function. Nature. (2008) 453:236–40. doi: 10.1038/nature06878 PMC2597437 18368049

[71] Xie H Sadim MS Sun Z. RORgammat recruits steroid receptor coactivators to ensure thymocyte survival. J Immunol. (2005) 175:3800–9. doi: 10.4049/jimmunol.175.6.3800 16148126

[72] Sen S Wang F Zhang J He Z Ma J Gwack Y. SRC1 promotes Th17 differentiation by overriding Foxp3 suppression to stimulate RORgammat activity in a PKC-theta-dependent manner. Proc Natl Acad Sci U.S.A. (2018) 115:E458–67. doi: 10.1073/pnas.1717789115 PMC5776998 29282318

[73] Ichiyama K Yoshida H Wakabayashi Y Chinen T Saeki K Nakaya M. Foxp3 inhibits RORgammat-mediated IL-17A mRNA transcription through direct interaction with RORgammat. J Biol Chem. (2008) 283:17003–8. doi: 10.1074/jbc.M801286200 18434325

[74] Tanaka K Martinez GJ Yan X Long W Ichiyama K Chi X. Regulation of pathogenic T helper 17 cell differentiation by steroid receptor coactivator-3. Cell Rep. (2018) 23:2318–29. doi: 10.1016/j.celrep.2018.04.088 29791844

[75] He Z Zhang J Du Q Xu J Gwack Y Sun Z. SRC3 is a cofactor for RORγt in Th17 differentiation but not thymocyte development. J Immunol. (2019) 202:760–9. doi: 10.4049/jimmunol.1801187 PMC6344289 30567733

[76] Sekiya T Kashiwagi I Inoue N Morita R Hori S Waldmann H. The nuclear orphan receptor Nr4a2 induces Foxp3 and regulates differentiation of CD4+ T cells. Nat Commun. (2011) 2:269. doi: 10.1038/ncomms1272 21468021 PMC3104557

[77] Chen T. Nuclear receptor drug discovery. Curr Opin Chem Biol. (2008) 12:418–26. doi: 10.1016/j.cbpa.2008.07.001 18662801

[78] Katti A Diaz BJ Caragine CM Sanjana NE Dow LE. CRISPR in cancer biology and therapy. Nat Rev Cancer. (2022) 22:259–79. doi: 10.1038/s41568-022-00441-w 35194172

[79] Zeng J Li M Zhao Q Chen M Zhao L Wei S. Small molecule inhibitors of RORγt for Th17 regulation in inflammatory and autoimmune diseases. J Pharm Anal. (2023) 13:545–62. doi: 10.1016/j.jpha.2023.05.009 PMC10334362 37440911

[80] Solt LA Kumar N Nuhant P Wang Y Lauer JL Liu J. Suppression of TH17 differentiation and autoimmunity by a synthetic ROR ligand. Nature. (2011) 472:491–4. doi: 10.1038/nature10075 PMC3148894 21499262

